# The non-neuronal cyclin-dependent kinase 5 is a fibrotic mediator potentially implicated in systemic sclerosis and a novel therapeutic target

**DOI:** 10.18632/oncotarget.23516

**Published:** 2017-12-20

**Authors:** Jun Wei, Roberta G. Marangoni, Feng Fang, Wenxia Wang, Jingang Huang, Joerg H.W. Distler, John Varga

**Affiliations:** ^1^ Northwestern Scleroderma Program, Department of Medicine, Feinberg School of Medicine, Chicago, IL, USA; ^2^ Department of Internal Medicine, University of Erlangen-Nuremberg and University Hospital Erlangen, Erlangen, Germany

**Keywords:** fibrosis, fibroblast, adipocyte, myofibroblast, CDK5

## Abstract

The mechanisms underlying persistent fibroblast activation and myofibroblast phenoconversion in underlying multi-organ fibrosis in systemic sclerosis (SSc) remain incompletely understood, hindering effective therapies to slow or reverse the process. Cyclin-dependent kinase 5 (CDK5) is a pleiotropic member of the CDK family originally identified in neuronal cells. In contrast to other CDKs, CDK5 activity depends on its CDK5R1 subunit p35. Here we demonstrate that expression of p35 and CDK5 activity are induced by TGF-ß in fibroblasts and adipocytic cell types. Levels of p35 are markedly elevated in both SSc skin biopsies and explanted SSc fibroblasts, as well as in fibrotic skin in mice. Ectopic p35 and CDK5 suppressed adipogenic markers while stimulating collagen production and myofibroblast markers, whereas RNAi-mediated CDK5 knockdown abrogated TGF-β fibrotic responses in a Smad-independent manner. Pharmacological inhibitors of CDK5 likewise prevented and reversed TGF-β responses in fibroblast monolayers and in *ex vivo* human skin organ cultures, ameliorated collagen overproduction in SSc fibroblasts, and prevented and reversed skin fibrosis in two distinct mouse models of SSc. Together, these results reveal a previously unrecognized key function for p35/CDK5 as a mediator of mesenchymal cell fibrotic responses. The results suggest a potential pathogenic role for elevated p35 expression and CDK5 activity in SSc, and raise the possibility that their selective pharmacological targeting might represent a novel treatment approach in fibrosis.

## INTRODUCTION

Systemic sclerosis (SSc) is associated with multi-organ fibrosis [1]. Skin fibrosis, a hallmark of SSc, is attributed to phenoconversion of tissue-resident mesenchymal progenitor cells to myofibroblasts, which produce extracellular matrix molecules, exert traction forces resulting in stiffness and extensive matrix remodeling, and resist apoptosis. The cellular origins of myofibroblasts in the fibrotic dermis remain to be fully elucidated. Studies indicate that a variety of mesenchymal progenitor cells, including pericytes and adipocytes may give rise to fibrotic myofibroblasts in response to transforming growth factor-ß (TGF-ß), Wnt ligands and developmental pathways, hypoxia, and substrate rigidity [2]. Impaired expression or function of the adipogenic master regulator PPAR-γ facilitates fibroblast activation and myofibroblast phenoconversion, and contributes to persistence of fibrosis in the skin [3–5]. While the processes underlying fibrosis generally recapitulate those driving normal wound healing, their temporal-spatial dysregulation culminates in non-resolving pathological scar rather than tissue regeneration. Thus, the factors that orchestrate wound healing and their deregulation resulting in non-resolving fibrosis are of substantial interest, and represent potential therapeutic targets [2, 6].

Cyclin-dependent kinase 5 (CDK5), a conserved member of the CDK family expressed principally in the central nervous system, is a pleiotropic non-receptor serine-threonine kinase implicated in angiogenesis, apoptosis, cell migration and senescence [7]. In light of its potent and wide-ranging biological activities and diverse intracellular substrates, CDK5 must be tightly regulated, and aberrant CDK5 activation is implicated in neurodegenerative diseases and cancer, and various disease processes in mouse models [8, 9]. CDK5 is an atypical cyclin-dependent kinase, since in contrast to other CDK family members, CDK5 activity requires the p35 (CDK5R1) subunit rather than cyclin. In light of recent studies linking aberrant CDK5 expression and activity with epithelial mesenchymal transition and renal fibrosis [10, 11], we hypothesized that in SSc patients, cutaneous TGF-ß hyperactivity in SSc might be associated with augmented CDK5 expression and activity, contributing to development and persistence of skin fibrosis. The present results show that p35 levels were markedly elevated in SSc skin biopsies, explanted SSc fibroblasts, and lesional skin from mice with experimentally induced fibrosis. *In vitro*, TGF-ß treatment up-regulated p35 expression and CDK5 activity in human and mouse skin fibroblasts, mesenchymal progenitor cells and adipocytes. In adipocytes, ectopic expression of p35 and CDK5 caused suppression of adiponectin synthesis while stimulating collagen. In fibroblasts, ectopic p35 elicited fibrotic responses whereas p35/CDK5 silencing abrogated stimulation of these responses by TGF-β. Moreover, pharmacological inhibitors of CDK5 prevented, as well as reversed, stimulation of collagen synthesis and myofibroblast differentiation in fibroblast monolayer cultures and in *ex vivo* skin organ cultures, ameliorated collagen overproduction in SSc fibroblasts, and prevented and reversed skin fibrosis in distinct *in vivo* models of SSc. These results suggest, for the first time, that deregulated CDK5/p35 signaling may have a pathogenic role in SSc fibrosis, and identify the TGF-ß-CDK5 axis as a potential novel target for therapy.

## RESULTS

### The p35 activator subunit of CDK5 is elevated in SSc and in murine scleroderma

**Figure 1:**
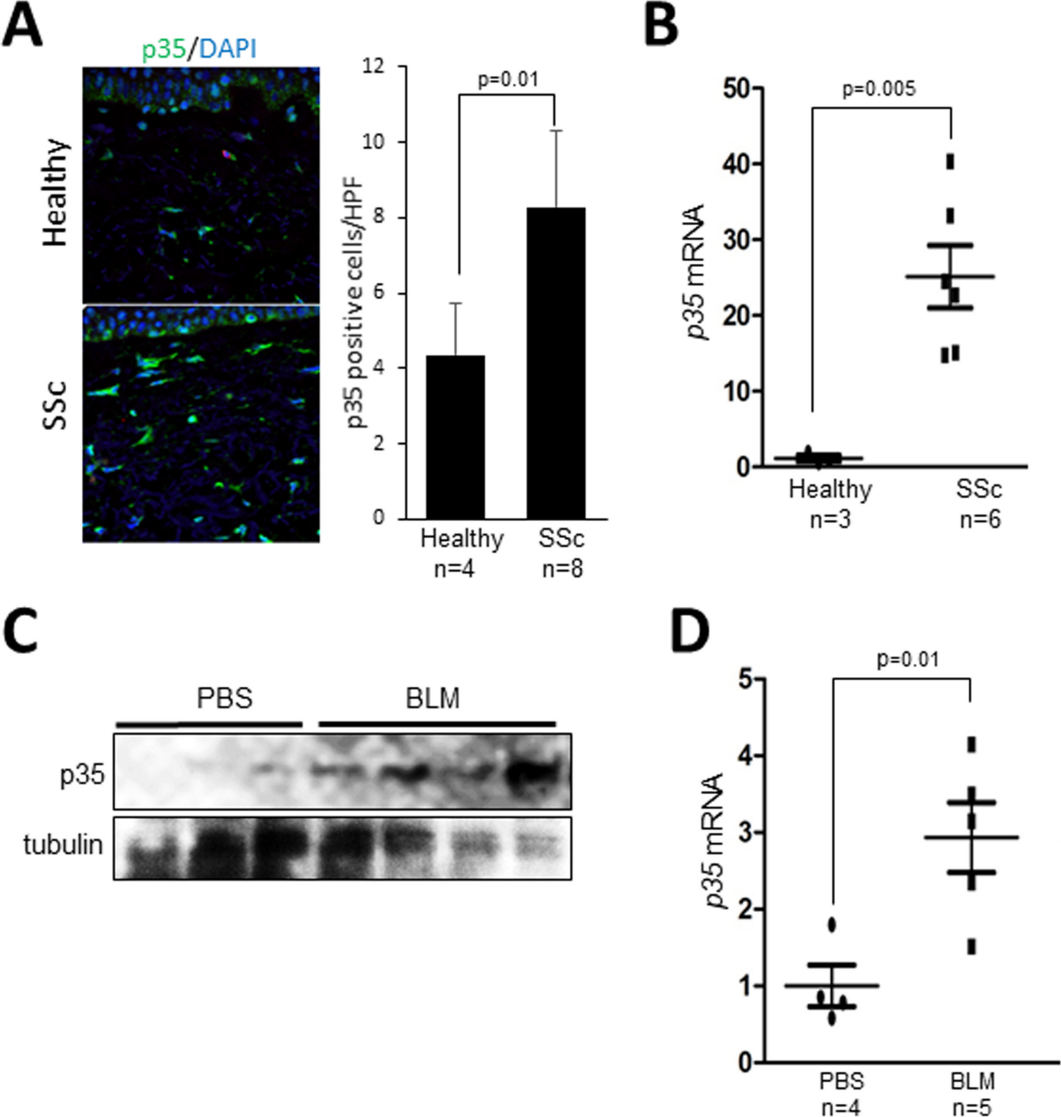
Elevated p35 expression in SSc skin biopsies (**A**) Immunofluorescence. Skin biopsies from SSc patients (*n* = 8) and healthy controls (*n* = 4) were immunostained with anti-p35 antibodies (green); nuclei were identified using DAPI (blue). Left panel, representative images; bar=20 μm. Right panels, quantitation of p35+ cells. Results are mean ± SEM; ^*^p < 0.05. (**B**) Real-time qPCR. RNA from healthy (*n* = 3) and SSc (*n* = 6) fibroblasts Results, normalized to GAPDH, represent mean of triplicate determinations from each cell line. ^*^p < 0.05. (**C**, **D**) p35 levels in bleomycin-induced SSc. Mice were given s.c. injections of bleomycin (*n* = 4-5) or PBS (*n* = 3-4) daily for 14 consecutive days, sacrificed on day 28, and skin harvested for Western analysis (C) and real-time qPCR; results, normalized to 36B4, are means ± SD of triplicate determinations from each skin biopsy. ^*^p < 0.05. BLM, bleomycin. Scale bar, 20 μm.

Immunohistology showed that p35 levels were markedly increased in SSc skin biopsies compared to healthy controls, with spindle-shaped p35-positive interstitial cells detected throughout the fibrotic dermis (Figure 1A). To examine the cell-autonomous expression of p35, skin fibroblasts derived from dcSSc patients (*n* = 6) and healthy controls (*n* = 3) were examined. Results of qPCR indicated that p35 mRNA levels were markedly elevated in SSc fibroblasts (*p* = 0.005; Figure 1B). Lesional skin from mice with bleomycin-induced SSc (*n* = 4–5) also showed increased p35 protein levels and p35 mRNA expression compared to PBS-treated control mice (*n* = 3–4) (Figure 1C, 1D).

### TGF-β up-regulates p35 expression and induces CDK5 activation

**Figure 2:**
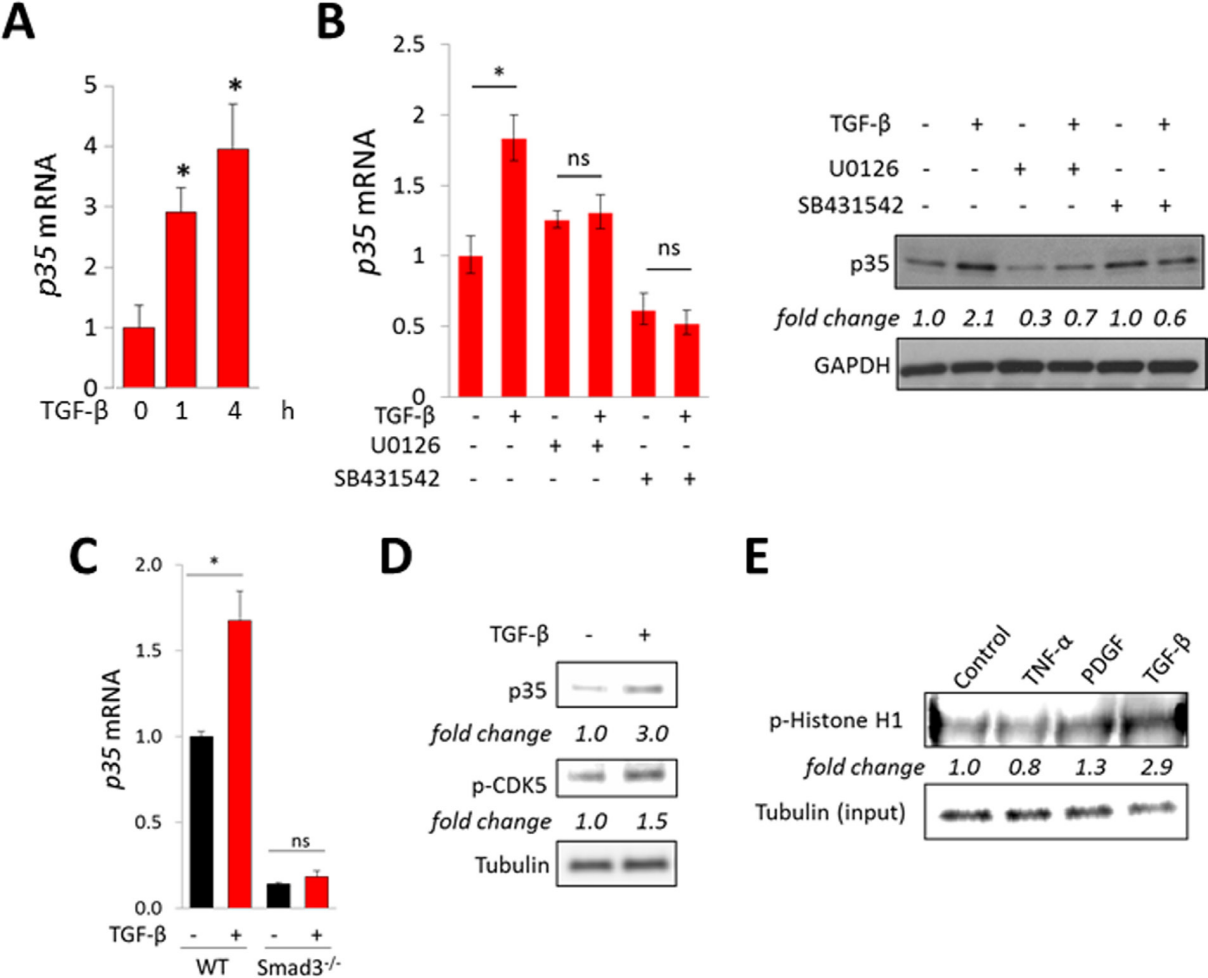
TGF-β stimulates p35 expression and CDK5 activation Confluent human foreskin (**A**–**E**) or mouse embryonic (C) fibroblasts were incubated in media with TGF-β (10 ng/ml). (A) Real-time qPCR. Results, normalized to GAPDH, represent means ± SD of triplicate determinations from an experiment representative of three. ^*^p < 0.05. (B) Cultures were pre-incubated with U0126 (10 μM) or SB431542 (5 μM) for 30 min followed by TGF-β incubation for 24 h. Left panel, real-time qPCR. Results, normalized to GAPDH, represent means ± SD of triplicate determinations from an experiment representative of three. ^*^p < 0.05. Right panels, Western analysis, representative images. (C) Smad3-null and wildtype MEFs were incubated with TGF-β for 4 h. Results of qPCR, normalized to GAPDH, represent means ± SD of triplicate determinations from an experiment representative of three. ^*^p < 0.05. (D) Foreskin fibroblasts were incubated with TGF-β for 60 min, and whole cell lysates were subjected to Western analysis. Representative images. Band intensities normalized to tubulin shown below. (E) Foreskin fibroblasts were incubated with TNF-α, PDGF or TGF-β for 60 min, whole cell lysates were immunoprecipitated with CDK5 antibodies, incubated with Histone H1 and phosphorylation examined using p-Histone H1 antibody. Band intensities normalized to input tubulin are shown below.

Since SSc skin biopsies show TGF-β pathway activation *in situ* [12], we sought to examine if TGF-ß might underlie up-regulation of p35 observed in these biopsies. Incubation of confluent skin fibroblasts with TGF-β resulted in dose- and time-dependent increase in the levels of p35 mRNA and protein (Figure 2A), while levels of CDK5 were unaffected (data not shown). Similar results were seen when human and mouse pre-adipocytes were incubated with TGF-ß (data not shown). The stimulation of p35 mRNA and protein elicited by TGF-β was abrogated in the presence of the Smad2/3 inhibitor SB431542 (Figure 2B). We further defined the role of Smad3 in p35 induction using fibroblasts deficient in Smad3, which in contrast to wildtype fibroblasts, failed to up-regulate p35 mRNA, or collagen gene expression, in response to TGF-β (Figure 2C). Because expression of p35 largely determines the level of CDK5 activity, we next investigated the effect of TGF-ß on CDK5 phosphorylation and activity. As shown in Figure 2D, stimulation of p35 in TGF-ß-treated fibroblasts was accompanied by increased CDK5 phosphorylation. Furthermore, nuclear extracts from TGF-β-treated cells demonstrated a three-fold increase in CDK5 activity (Figure 2E). Taken together, these results indicate that the CDK5 activator subunit p35 is elevated in human and mouse skin fibrosis, its expression is induced by TGF-ß in a Smad-dependent manner, and it mediates enhanced CDK5 activity in TGF-ß-treated fibroblasts.

### p35 suppresses adiponectin production in adipocytic cells and stimulates profibrotic gene expression

**Figure 3:**
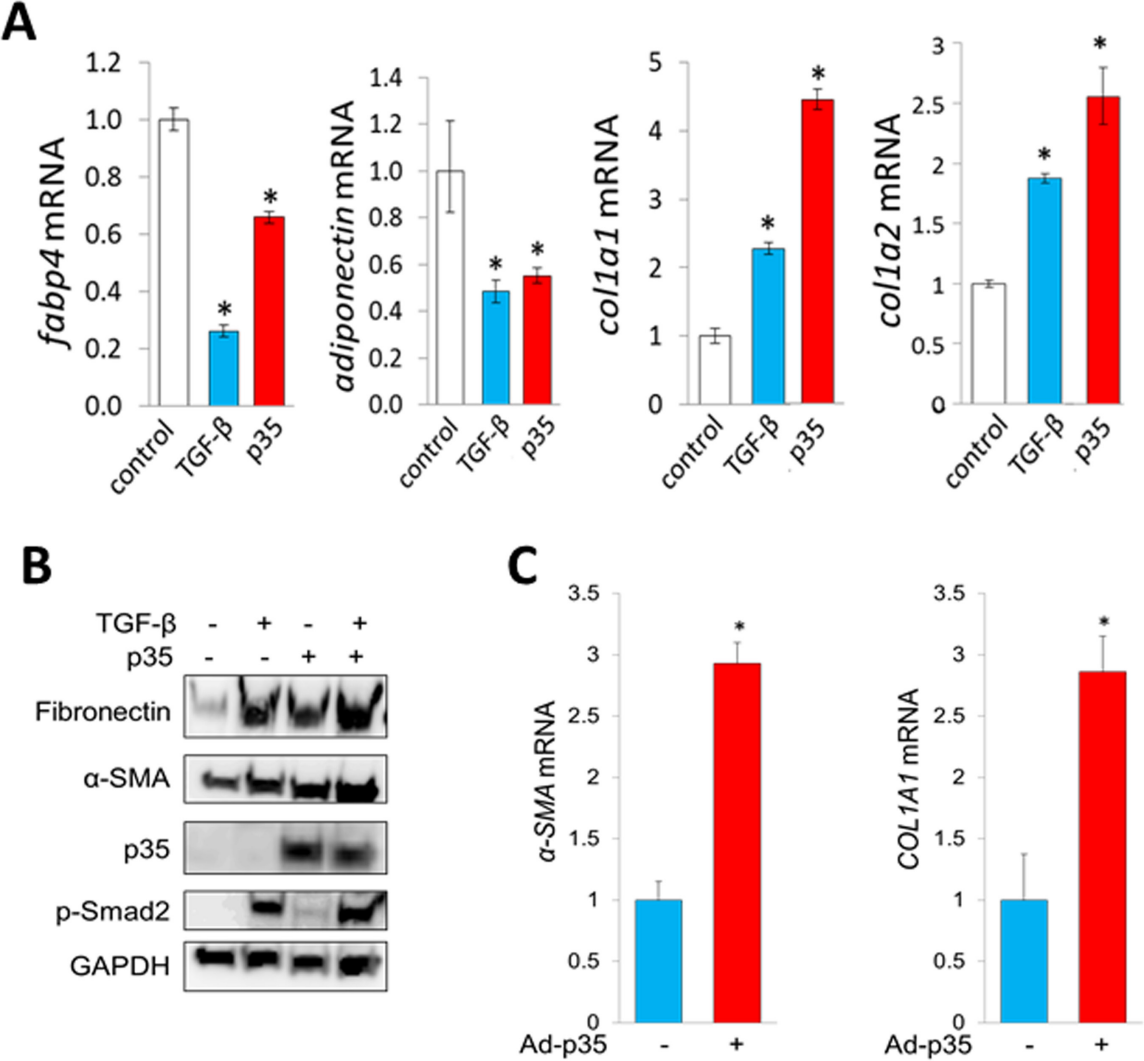
p35 has profibrotic effects in mesenchymal cells (**A**) Real-time qPCR. Mouse 3T3-L1 preadipocytes were incubated with differentiation media for 2 days followed by maintenance media for 3 days, transfected with p35 and incubated with TGF-β (10 ng/ml) for a further 48 h. Results, normalized to *36B4*, represent means ± SD of triplicate determinations from an experiment representative of three. ^*^p < 0.05. (**B**, **C**) Foreskin fibroblasts were infected with Ad-p35 (30 MOI) and incubated with TGF-β for 72 h. (B) Western analysis. Representative images. (C) Real-time qPCR results, normalized to GAPDH, represent means ± SD of triplicate determinations from an experiment representative of three. ^*^p < 0.05.

Tissue-resident adipogenic cells represent a principal source of mesenchymal progenitors giving rise to differentiated myofibroblasts in skin fibrosis [13]. Lineage determination of preadipocytic cells toward an adipogenic or fibroblast-like cell fate is largely governed by PPAR-γ. Recent studies show that PPAR-γ activity can be modulated by CDK5-dependent phosphorylation (34). To examine the potential contribution of CDK5 in fibrotic cellular responses, we first investigated the effect of CDK5 in adipocytic cells using a gain-of-function strategy. Ectopic expression of CDK5 and p35 in adipocytic cells both caused marked suppression of PPAR-γ along with its target genes, while at the same time inducing stimulation of type I collagen gen expression (Figure 3A). Human skin fibroblasts infected with adenoviral p35 showed increased expression of fibronectin and αSMA (Figure 3B, 3C).

### Inhibition of CDK5 ameliorates TGF-β-induced profibrotic responses

**Figure 4:**
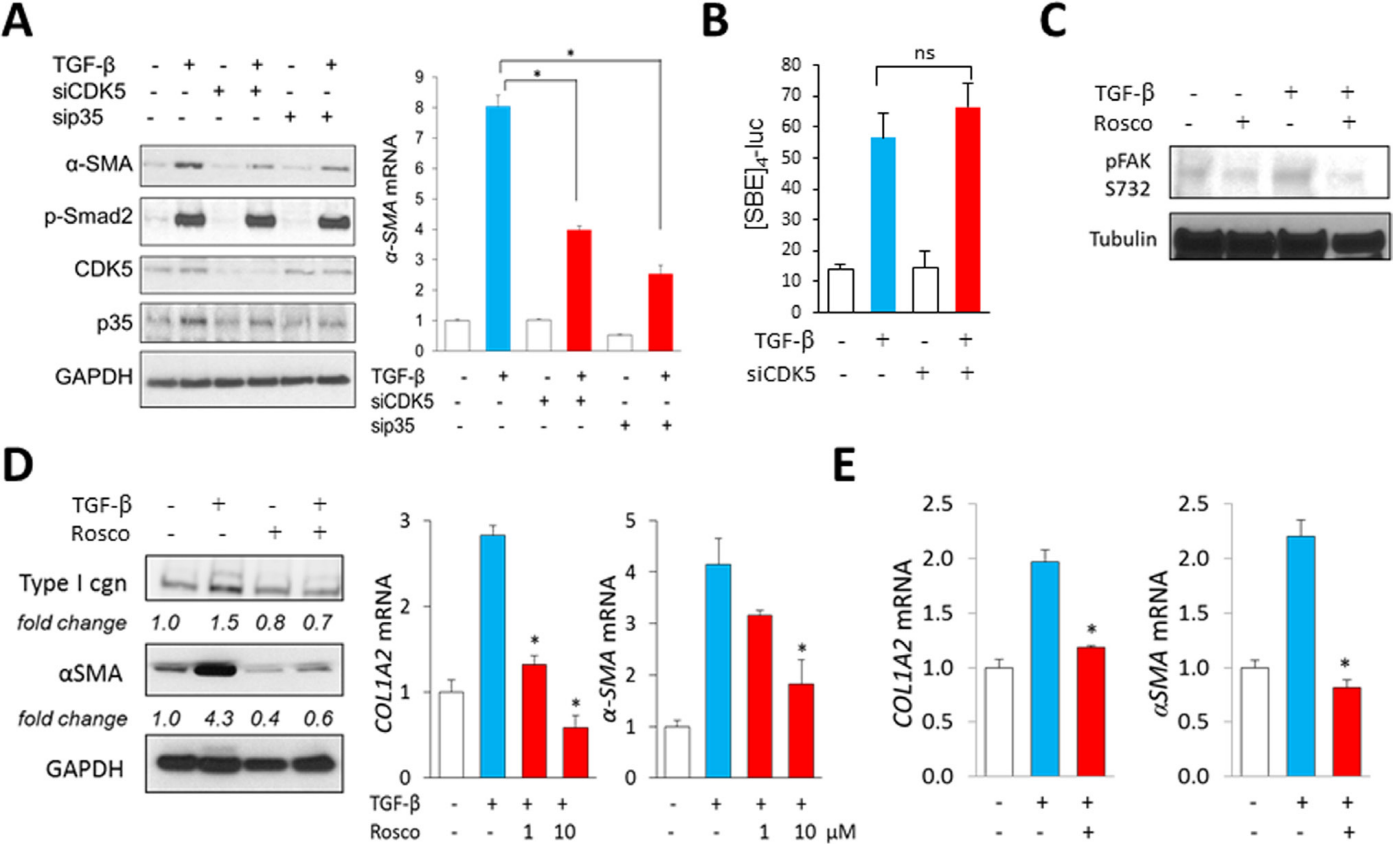
Inhibition of CDK5 signaling in fibroblasts abrogates TGF-β-induced responses (**A**) Fibroblasts transfected with indicated siRNA for 24 h followed by incubation with TGF-β2 (10 ng/ml) for further 24 h. Left panels, Western analysis. Representative images. Right panel, real-time qPCR. Results, normalized with GAPDH, are mean ± SD of triplicate determinations from an experiment representative of three. ^*^p < 0.05. (**B**) Fibroblasts with CDK5 siRNA were transiently transfected with [SBE]4-luc, followed by TGF-β (10 ng/ml) for 24 h. Whole cell lysates were assayed for their luciferase activities. Results, normalized with renilla luciferase, represent the means ± SD from triplicate experiments. ^*^p < 0.05. (**C**) Fibroblasts were incubated in media with roscovitine (10 μM) and TGF-β for 30 min, and whole cell lysates were examined by Western analysis. Representative images. (**D**) Fibroblasts were incubated with roscovitine (10 μM) for 30 min followed by incubation with TGF-ß (10 ng/ml) for 24 h. Left panels, Western analysis. Representative images. Right panel, real-time qPCR. Results, normalized with GAPDH, are mean ± SD of triplicate determinations from an experiment representative of three. ^*^ *p* < 0.05. (**E**) Fibroblasts were preincubated with TGF-ß for 24 h, followed by roscovitine (10 μM) for 24 h. Real-time qPCR results, normalized with GAPDH, are mean ± SD of triplicate determinations from an experiment representative of three. ^*^p < 0.05.

**Figure 5:**
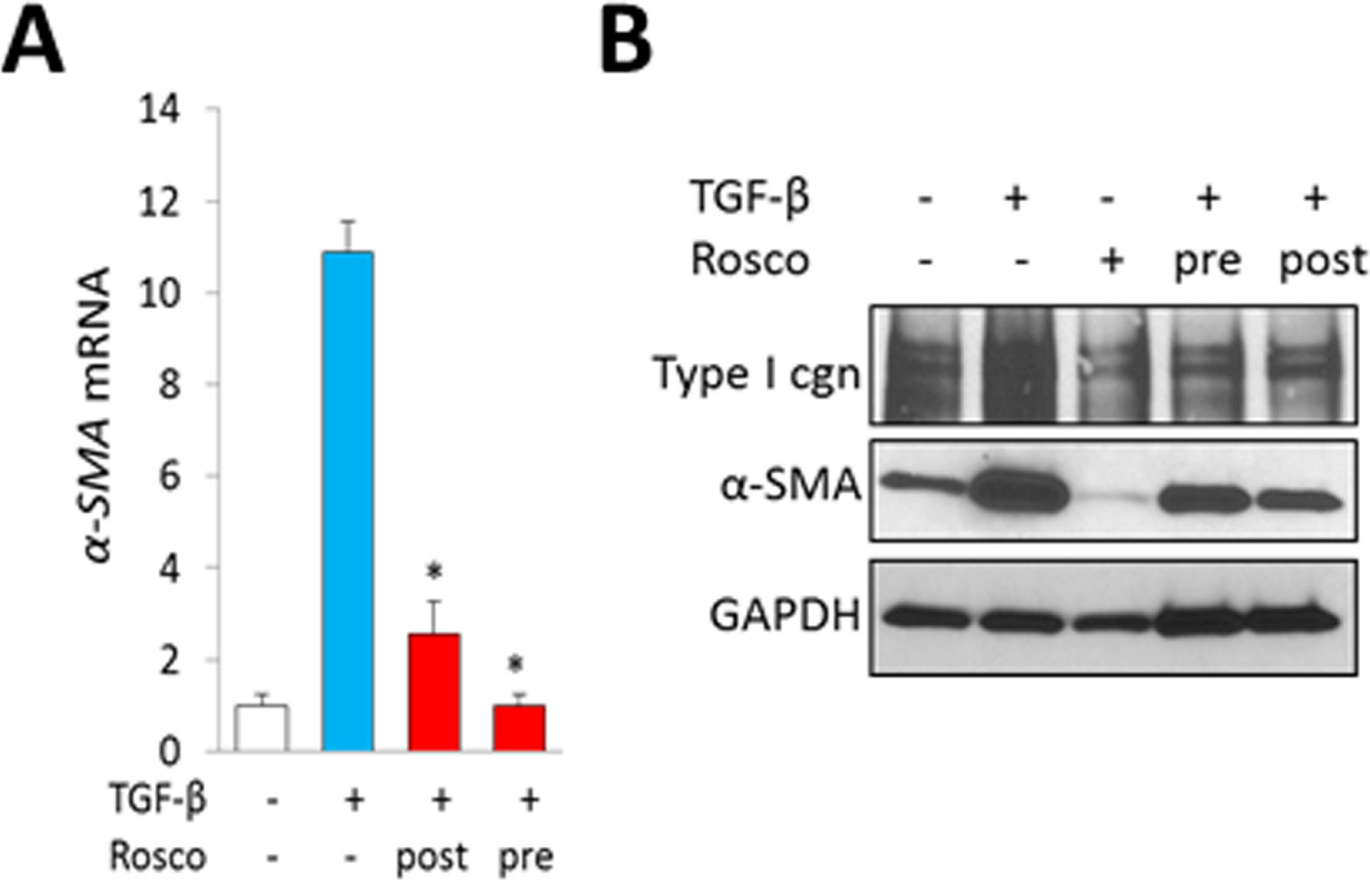
Inhibition of CDK5 abrogates TGF-ß responses in skin organ cultures Skin explants were incubated with TGF-β (10 ng/ml) for 6 d, with roscovitine (10 μM) added to the media 30 min before (pre-treatment) or 48 h following (post-treatment) TGF-β. (**A**) Real-time qPCR results, normalized with GAPDH, are means ± SD of triplicate determines from a representative experiment. ^*^p < 0.05. (**B**) Western analysis. Representative images.

Orthogonal approaches were used to identify the impact of CDK5 on profibrotic responses. First, we found that knockdown of either CDK5 or p35 in fibroblasts was sufficient to abrogate αSMA stimulation by TGF-ß, while ligand-induced Smad2 phosphorylation remained largely unaffected in these cells (Figure 4A). Moreover, transient transfection assays showed that stimulation of the Smad-responsive [SBE]_4_-luc reporter by TGF-β was likewise largely unaffected by CDK5 knockdown (Figure 4B). Second, treatment of fibroblasts with the CDK5-specific inhibitor roscovitine significantly reduced histone H1 phosphorylation elicited by TGF-ß, consistent with inhibition of CDK5 (data not shown). Consistent with the results from CDK5/p35 knockdown experiments, Smad2 phosphorylation was largely unaffected by roscovitine (Supplementary Figure 1). In contrast, TGF-ß-induced activation of FAK, which drives focal adhesion complex assembly essential for mediating fibrotic responses, was reduced in roscovitine-treated fibroblasts (Figure 4C). Pre-incubation of the cultures with roscovitine was noted to suppress TGF-β–dependent fibrotic responses in a dose-dependent manner (Figure 4D). Importantly, roscovitine was capable of reversing stimulation of collagen and α-SMA expression even when it was added to the cultures up to 24 h following TGF-β (Figure 4E). To further characterize the effects of roscovitine, we used human skin explants stimulated *ex vivo* with TGF-ß. Both pre- and post-treatment with roscovitine attenuated the induction of collagen and αSMA elicited by TGF-β (Figure 5A, 5B). Comparable anti-fibrotic effects were elicited by treatment of the explants with SCH727965 (dinaciclib), a potent but relatively non-selective CDK5 inhibitor (data not show). Together, these results indicate that CDK5 activity is both necessary and sufficient for eliciting fibrotic responses in mesenchymal cells.

### Roscovitine treatment normalizes collagen and αSMA expression in SSc fibroblasts

**Figure 6:**
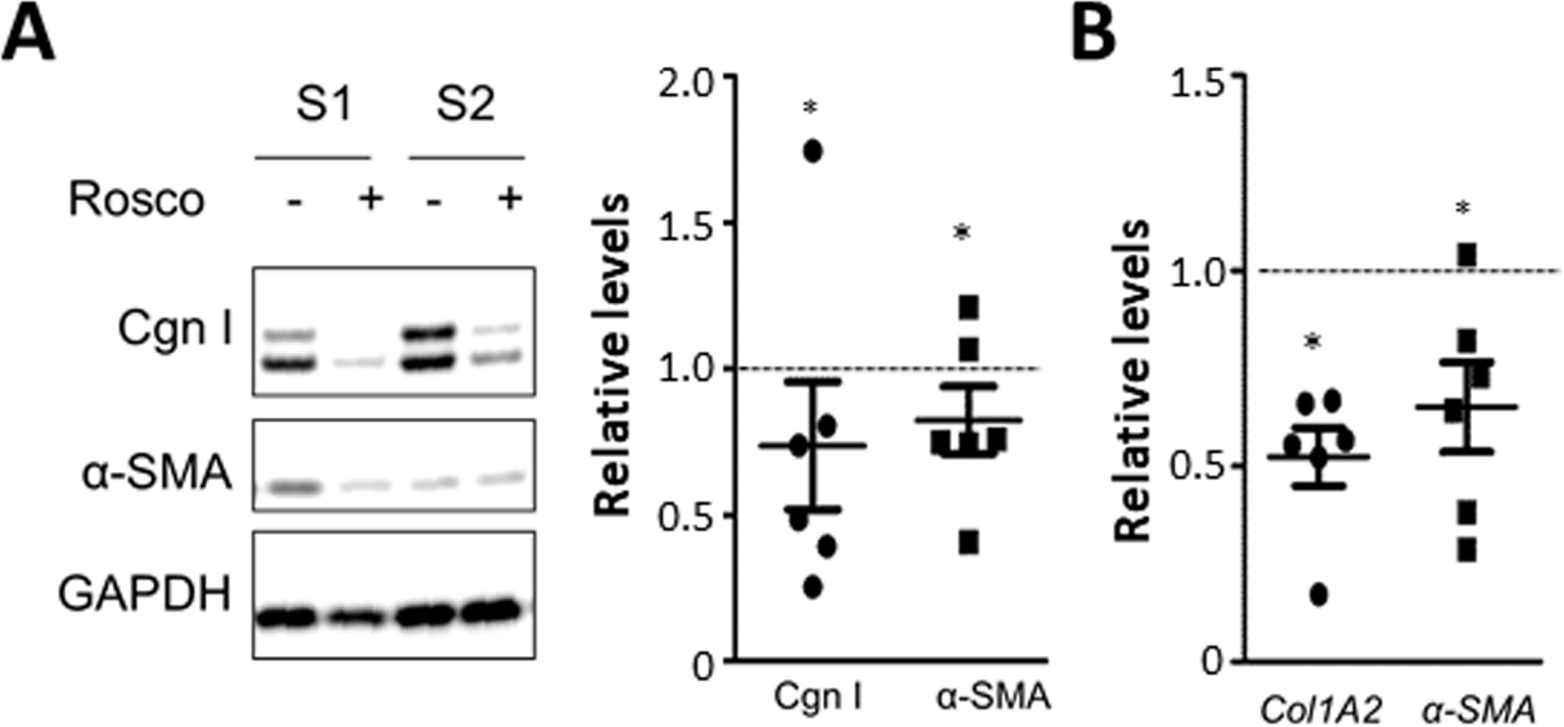
Roscovitine normalizes collagen overexpression in SSc fibroblasts SSc skin fibroblasts (*n* = 6) were incubated with roscovitine (10 μM) for 48 h. (**A**) Western analysis of whole cell lysates and culture supernatants. Left panels, Representative images. Cgn I, Type I collagen. Right panel, band intensities normalized with GAPDH levels expressed relative to untreated cultures. (**B**) Real-time qPCR. Results are expressed as dot plots of mRNA levels relative to untreated cultures.

We and others had previously demonstrated that explanted SSc fibroblasts maintained their activated phenotype *ex vivo* even in the absence of exogenous ligand [14]. We found that the expression of p35 was increased in SSc fibroblasts (Figure 1B). Treatment of SSc fibroblasts (*n* = 6) with roscovitine resulted in 20–30% reduction (*p* < 0.05) in cellular Type I collagen and αSMA levels (Figure 6A), and >40% reduction in *COL1A2* mRNA (Figure 6B).

### Roscovitine treatment attenuates fibrosis in distinct mouse models of systemic sclerosis

**Figure 7:**
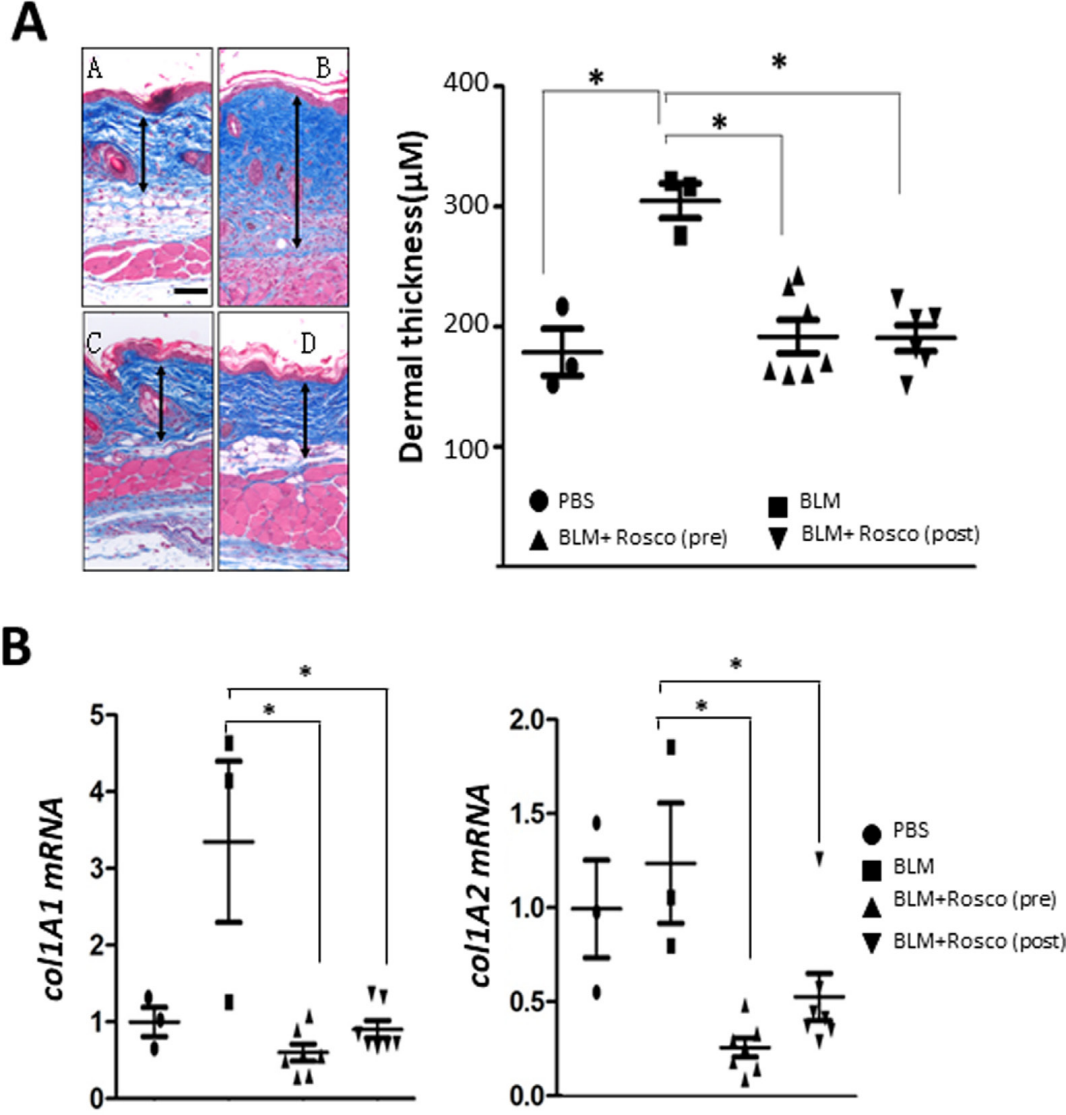
Roscovitine ameliorates and reverses bleomycin-induced scleroderma Mice given s.c. bleomycin daily for 14 days, together with Roscovitine (50 mg/kg i.p.) daily started concurrently with, or on day 15, of bleomycin, and sacrificed at day 28. Lesional skin was harvested. (**A**) Left panels, Trichrome stain (original magnification 200 x). Arrows delineate the dermis. Right panel, dermal thickness; means ± SD of 5 determinations/hpf from 3-7 mice/group. Scale bar, 50 μm. (**B**) Real-time qPCR. Results, normalized with *36b4*, represent the means ± SD of triplicate determinations from 3-7 mice/group. ^*^ *p* < 0.05.

**Figure 8:**
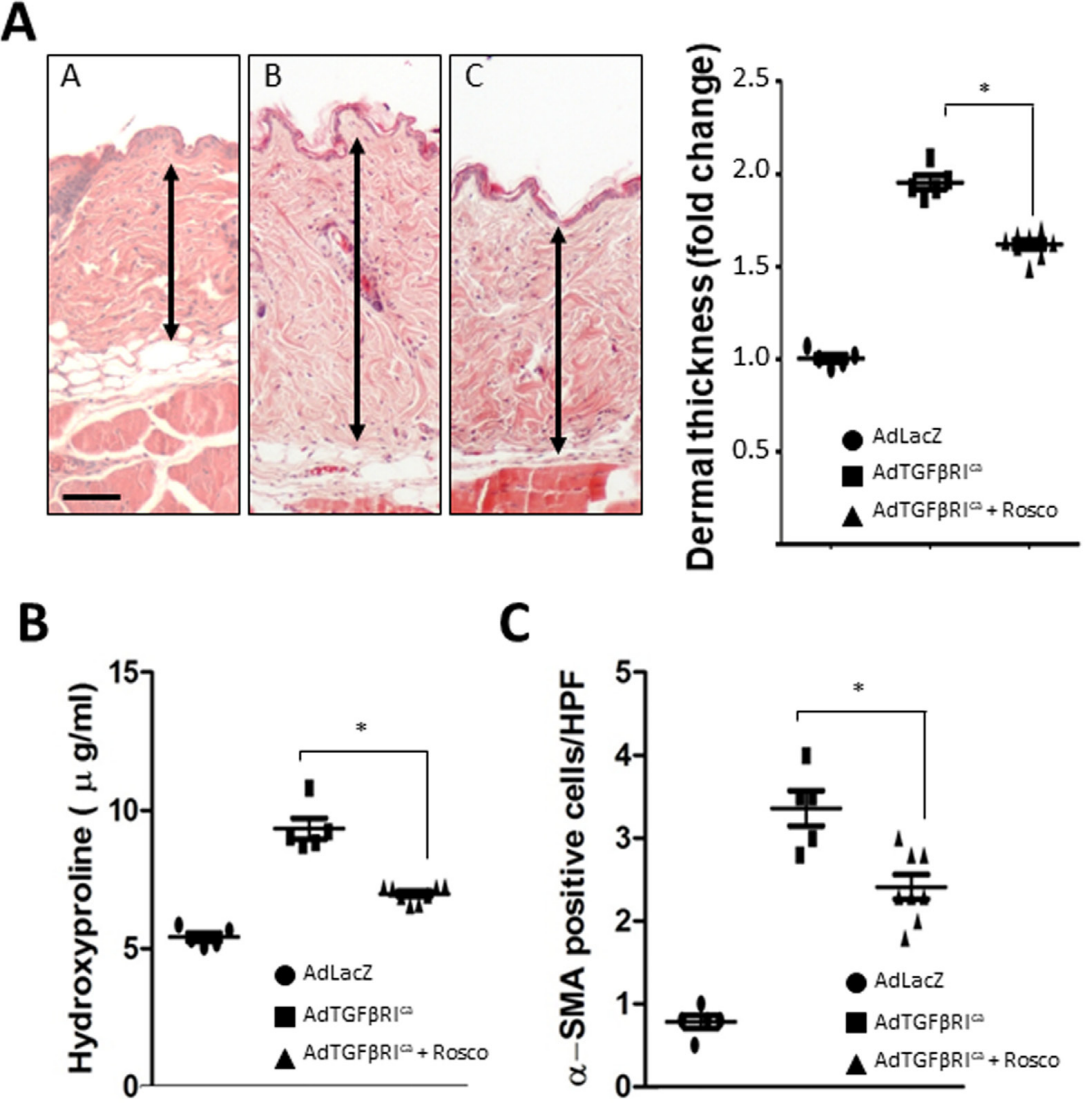
Roscovitine ameliorates TβRI-induced skin fibrosis Mice were given i.c. Ad-TβRI^ca^ or Ad-lacZ twice along with daily i.p. roscovitine (50 mg/kg). At the end of the experiments, lesional skin was harvested for analysis. (**A**) Left panels, hematoxylin and eosin stain (original magnification 200×). Right panel, dermal thickness. Results, representing means ± SEM of 6-8 mice/group, shown as -fold change compared to control. Scale bar, 50 μm. (**B**) Hydroxyproline assays. The results, representing means ± SEM of triplicate determinations from 6-8 mice/group, are shown as -fold change compared to control. (**C**) α-SMA+ cells in the dermis were identified by immunohistochemistry. Results are means ± SEM. ^*^ *p* < 0.05.

In light of the Smad-independent pro-fibrotic effects of CDK5, we sought to evaluate the impact of its pharmacological inhibition *in vivo* in complementary fibrotic disease models. In the first approach, skin fibrosis was induced in C57BL/6J mice by daily s.c. bleomycin injections. Mice were given concurrent roscovitine (50 mg/kg) or vehicle by i.p. injections starting either on day 1 (prevention), or day 15 (reversal) of bleomycin, and sacrificed on day 28. Long-term roscovitine administration was well tolerated, and no significant weight loss or other signs of toxicity were noted. Significant increase in the thickness of the dermis was seen in mice injected with bleomycin compared to PBS (307±17 vs.182±19 μm). Enhanced collagen deposition, increased dermal thickness, and up-regulation of Type I collagen, were all markedly ameliorated when roscovitine was administered starting concurrently with bleomycin, or on day 15 (Figure 7 and Supplementary Figure 2). In a complementary experimental approach, C57BL/6J mice were given two i.c. injections of Ad-TβRI^ca^ or Ad-LacZ separated by four weeks, along with roscovitine or vehicle by daily i.p. injections, and sacrificed four weeks following the second adenovirus injection. TβRI^ca^ elicited robust TGF-β activity in the lesional skin that was associated with significant dermal thickening, increased collagen deposition, and myofibroblast accumulation (Figure 8). Concomitant treatment of the mice with roscovitine significantly abrogated all of these fibrotic responses.

## DISCUSSION

The factors responsible for persistent fibroblast activation underlying pathological fibrosis in SSc remain incompletely characterized. A number of recent studies have focused attention on CDK5, an atypical cyclin-dependent kinase originally identified in the CNS but shown to be ubiquitously expressed in tissues, and increasingly implicated in a variety of physiological and pathological processes [7]. Unlike other members of the CDK family, CDK5 does not require binding to cyclins for its activity. Instead, CDK5 is activated by interacting with its regulatory p35 subunit. The present studies indicate that the p35 is up-regulated in lesional skin from patients with SSc, and from mice with experimentally-induced skin fibrosis. Moreover, we show by genetic and pharmacological approaches that p35 and CDK5 mediate key effects elicited by TGF-ß, and are necessary and sufficient for fibrotic responses in mesenchymal cells. Treatment of mice with the CDK5 inhibitor roscovitine prevented and reversed dermal fibrosis induced by bleomycin or by TGF-ß receptor. Together, our findings provide the first evidence implicating aberrant CDK5 function in fibrosis in SSc, and suggest that pharmacological targeting of the CDK5 pathway may represent a potential therapeutic strategy for fibrosis.

Fibrosis of the skin and internal organs is the hallmark of SSc, and represents a deregulated healing process [6]. TGF-ß is recognized to play a key role in triggering transcriptional programs responsible for the activated fibroblast phenotype in SSc and other fibrotic disorders [2]. Furthermore, TGF-ß appears to be a central factor in promoting the emergence of activated myofibroblasts originating from diverse tissue-resident populations of mesenchymal progenitor cells. In particular, recent studies have indicated that dermal white adipose tissue contains adipocytic cells that differentiate into mature adipocytes, or alternately show a cell fate switch and differentiate into myofibroblasts [5, 15, 16]. Dermal adipocytic cell differentiation is controlled by PPAR-γ, a master regulator of adipogenesis. We had demonstrated that TGF-ß represses PPAR-γ expression and function in adipocytes, which may directly and indirectly contribute to preferential myofibroblast differentiation of these cells in dermal fibrosis [3, 5, 13]. Fibrotic responses elicited by TGF-ß, including up-regulation of collagen gene expression and αSMA-positive stress fiber formation, are mediated via canonical (Smad-dependent) intracellular signaling. However, the Smad-independent signaling pathways also contributing to fibrotic responses, and the role of hypoxia and additional non-TGF-ß-dependent events implicated in development and persistence of fibrosis, remain incompletely characterized.

Cyclin-dependent kinase 5 (CDK5), a highly conserved member of the CDK family, is a non-receptor serine-threonine kinase with essential roles in a variety of cell processes [7]. In contrast to other CDKs, CDK5 is activated in a cyclin-independent manner by bindings to the p35 activator subunit [17]. In response to hypoxia, inflammatory signals and a variety of other stimuli, p35 itself can be cleaved to p25, which in turn causes hyperactivation of CDK5 signaling [18]. Originally described to play key roles in the neuronal development and CNS homeostasis, CDK5 has now been shown to be ubiquitously active in non-neuronal tissues, and to play key roles in a range of physiological processes [19]. These include angiogenesis, cell migration, inflammation and metabolism, while deregulated CDK5 signaling is implicated in neurodegeneration and Huntington's disease, as well as cancer [8, 9]. Of particular current interest is the emerging role of CDK5 in obesity, since PPAR-γ, the essential master regulator of adipogenesis required for mature adipocyte differentiation adipokine production [20, 21], was recently found to be a major substrate for CDK5-mediated phosphorylation and inactivation [22, 23]. Obesity is associated with elevated CDK5 activity, and resulting constitutive PPAR-γ phosphorylation and reduced activity contribute to impaired adipocyte maturation and reduced adiponectin secretion [22, 23].

These observations led us to hypothesize that the CDK5/p35 pathway might be responsible for impaired PPAR-γ function in SSc and thereby contribute to the development and persistence of fibrosis. We found that levels of p35 were markedly elevated in SSc skin biopsies and explanted SSc fibroblasts, as well as in lesional skin from mice with bleomycin-induced SSc. *In vitro*, both p35 expression and CDK5 activity were strongly stimulated by TGF-β in human and mouse skin fibroblasts, mesenchymal progenitor cells and mature adipocytes. Ectopic p35 and CDK5 caused simultaneous suppression of adiponectin expression and stimulation of collagen synthesis in these cells, whereas knockdown of p35/CDK5 abrogated TGF-β-induced fibrotic gene expression, without altering Smad activity. Pharmacological inhibitors of CDK5 prevented and reversed TGF-β-induced stimulation of collagen synthesis and myofibroblast differentiation in fibroblast-monolayer cultures and in *ex vivo* skin organ cultures, ameliorated collagen overproduction in SSc fibroblasts, and prevented and reversed skin fibrosis in complementary inflammatory and TGF-ß-driven mouse models of SSc. The anti-fibrotic effects of CDK5 blockade were independent of Smads, and were associated with reduced activation of FAK, an essential mediator of pathological fibrosis in SSc [6]. Previous studies implicated FAK as a CDK5 target potentially responsible for mediating its profibrotic effects. In neuronal cells CDK5 can cause FAK activation via phosphorylation on Ser732 [24]. These results implicate deregulated CDK5/p35 signaling in the pathogenesis of fibrosis in SSc, and identify this axis as a potential target for therapy.

In summary, our present results show that TGF-ß elicits CDK5 activation in fibroblasts via Smad-dependent up-regulation of p35 activator subunit. In light of the prominent pathogenic role of TGF-ß in SSc organ fibrosis, its stimulatory effect might provide an explanation for the elevated p35 expression observed in SSc skin biopsies. Moreover, similar stimulation of p35 and CDK5 activity has been observed with hypoxia, also prominent in fibrotic tissue, and might represent an additional mechanism accounting for up-regulation of p35 and CDK5 in SSc [18]. CDK5 activation is itself sufficient to elicit profibrotic responses in fibroblasts, and to reprogram adipocytic cells into collagen-producing fibroblasts (Supplementary Figure 3). The profibrotic effects of TGF-ß are partially dependent of CDK5 activity, and genetic and pharmacological targeting CDK5 attenuates the fibrotic process both *in vitro* and in mouse models. A recent study implicated CDK5 in renal fibrosis, and elevated p35 was shown to be predictive of fibrosis severity in diabetic nephropathy [11]. A variety of strategies have been developed for interrupting pathological CDK5 activity, and are in clinical trials in cancers [7]. Roscovitine was shown to mitigate the fibrotic phenotype of SSc fibroblasts in culture, consistent with our present findings [25]. Because CDK5 appears to function downstream of Smad2/3 in the TGF-ß-dependent fibrotic pathway, selective blockade of CDK5 activity might be expected to attenuate fibrotic responses, as demonstrated by our present findings, without disrupting other, potentially homeostatic, Smad-dependent TGF-ß responses, thus providing an advantageous selective therapeutic strategy. Our novel findings suggest an essential role for CDK5 in the pathogenesis of fibrosis by promoting adipocyte-fibroblast phenoconversion and activating a Smad-independent fibrotic program in fibroblasts. In light of these findings, treatments selectively targeting deregulated CDK5 activity in SSc should be evaluated in clinical trials.

## MATERIALS AND METHODS

### Cell cultures

Primary cultures of human fibroblasts were established by explantation from neonatal foreskin or from skin biopsies from the dorsal forearm of six healthy adults and six patients with diffuse cutaneous SSc (dcSSc) [26]. Skin biopsies were performed upon informed consent and in compliance with Northwestern University Institutional Review Board for Human Studies. Clinical characteristics of the patients are shown in Table 1.

**Table 1:** Clinical features of SSc subjects undergoing skin biopsy for immunofluorescence analysis

	Age (yrs)	Sex	MRSS	Skin score at biopsy site	Disease duration time of biopsy (months)
SSc 1	49	F	3	0	44
SSc 2	51	M	48	3	9
SSc 3	50	F	24	2	101
SSc 4	34	F	32	2	40
SSc 5	57	F	11	1	64
SSc 6	50	F	9	1	8
SSc 7	58	F	3	0	45
SSc 8	55	F	7	0	116

All skin biopsies were obtained from the forearm. MRSS, modified Rodnan Skin score (maximum 51). Biopsy site skin scores are as follows: 0, normal; 1, mild skin thickening; 2, moderate thickening; 3, severe thickening.

Human cells were maintained in Dulbecco's Modified Eagle's Medium (DMEM) (BioWhittaker, Walkersville, MD), and studied between passages 4–8 [3]. Mouse 3T3-L1 preadipocytes (from ATCC) were grown in Maintenance Medium (Zen-Bio, Research Triangle Park, NC), and differentiated to adipocytes as described [27]. At early confluence, fresh media containing indicated concentrations of the CDK5 inhibitor roscovitine (Cayman Chemical, Ann Arbor, MI) or human recombinant TGF-β2 (Peprotech, Rocky Hill, NJ) were added to the cultures and incubation continued for further 48 h. In selected experiments, cultures were pretreated with U0126 or SB431542 (both from Sigma, St. Louis, MO). At the end of the incubation periods, cultures were harvested and fibrotic responses evaluated by real-time qPCR, Western analysis and luciferase assays, as indicated. Cytotoxicity was evaluated using LDH cytotoxicity assay kits (Biovision, Mountain View, CA) and by Trypan blue dye exclusion.

### Human skin organ cultures

Fresh foreskin tissues were cut into 0.5 cm x 0.5 cm pieces containing complete epidermal and dermal layers, and were cultured in an air-medium interface with the epidermal and keratin layers side up and exposed to air. Media (DMEM) were replaced every other day with fresh media supplemented with 10% FBS, and cultures were incubated with TGF-β2 (10 ng/ml) for six days, with roscovitine (10 μM) added either 30 min before, or 48 h following, TGF-β2.

### RNA isolation and quantitative real-time polymerase chain reaction (qPCR)

Total RNA was isolated from cultured cells or mouse skin using Quick RNA^TM^ Miniprep (Zymo Research, Irvine, CA), and reverse transcribed for real-time qPCR using SuperScript First-strand synthesis system (Invitrogen, Carlsbad, CA). Real-time qPCR reactions were performed on an ABI-Prism 7300 sequence detection PCR machine (Applied Biosystem, Forster City, CA) [28]. mRNA expression levels were determined by calculating ΔΔC_t_ and normalized with *Gapdh* or *36B4* levels in each sample.

### Western analysis

At the end of the incubation periods, cultures were harvested, and equal amounts (5–15 μg) of whole cell lysates were subjected to electrophoresis in tris–glycine 4–15% gradient gels [28]. Membranes were incubated with the following primary antibodies: Type I collagen (1:400, Southern Biotechnology, Birmingham, AL), phospho-Smad2, phospho-FAK^S732^, phospho-histone H1, phospho-CDK5 (1:1000, Cell Signaling Technology, Danvers, MA), total CDK5, Smad1/2/3, p35 (1:200, Santa Cruz Biotechnology, Santa Cruz, CA), and GAPDH (1:3000, Invitrogen), followed by appropriate secondary antibodies. Antigen–antibody complexes were visualized by chemiluminescence (Pierce Biotechnology, Rockford, IL). ImageJ software (http://rsb.info.nih.gov/ij/) was used to measure band intensities.

### Transient transfection assays

Fibroblasts at early confluence were transiently transfected with [SBE]_4_-TK-luc reporter constructs or appropriate empty vectors [29] using Lipofectamine^TM^ LTX (Invitrogen, Carlsberg, CA). Following incubation with roscovitine and TGF-β for 24 h, cultures were harvested and whole cell lysates were assayed for their luciferase activities using the dual-luc reporter assay system (Promega, Madison, WI) [30]. pRL-TK Renilla luciferase (pRL-TK-Luc) was used in each experiment as an internal control and experiments were performed in triplicate.

### Animal experiments

Animal protocols were institutionally approved by the Animal Care and Use Committee of Northwestern University or the University of Erlangen-Nuremberg. Skin fibrosis was induced by two distinct approaches: subcutaneous (s.c.) bleomycin, or intracutaneous (i.c.) adenovirus expressing constitutively-active mutant Type I TGF-β receptor (TβRI^ca^). In the first protocol, six- to eight-week-old female C57BL/6J mice (Jackson Laboratory, Bar Harbor, ME) were given s.c. bleomycin (10 mg/kg) daily for 14 consecutive days, and sacrificed on day 28. In the second protocol, replication-deficient type 5 adenovirus encoding TβRI^ca^ or LacZ (6.7 × 10^7^ pfu/mouse) was injected i.c. on two occasions separated by four weeks, and mice were sacrificed four weeks following the second injection [31]. In both protocols, roscovitine (50 mg/kg, dissolved in 80% PBS, 10% DMSO, and 10% Cremophor-EL, Sigma, St. Louis, MO) was given daily by intraperitoneal (i.p.) injections. Lesional skin was harvested and analyzed as previously described [32].

### Immunocytochemistry

Fibroblasts were pretreated with roscovitine (5 μM) for 15 min, followed by incubation in media with 10 ng/ml TGF-β2. Twenty-four h later, cells were fixed in 4% paraformaldehyde, washed in phosphate buffered saline (PBS), and incubated with primary antibodies against αSMA (1: 400 dilution, Abcam) or Type I collagen (1:200, Southern Biotechnology) for 120 min, followed by Alexa^®^ 594-conjugated secondary antibodies (Invitrogen) for 60 min. Nuclei were identified by 4›-6-diamidino-2-phenylindole (DAPI) staining. Non-immune IgG was used as a negative control in each experiment. Following stringent washing, slides were examined under a Zeiss UV Meta 510 confocal microscope (Carl Zeiss, Jena, Germany). Each experiment was repeated at least three times with consistent results.

### Chemical, morphological, histochemical and immunohistochemical analyses of skin

Consecutive 4-μm serial sections of paraffin-embedded mouse skin were stained with hematoxylin and eosin (H&E) or Trichrome [33]. Dermal thickness, defined as the distance between the epidermal-dermal and dermal-intradermal adipose junctions, was determined at five randomly selected locations/slide for each mouse [18]. Total collagen content in skin samples was determined by hydroxyproline assays [34]. For quantification of myofibroblasts, slides were immunostained with anti-αSMA antibodies (Sigma-Aldrich) followed by biotinylated secondary antibodies (all Vector labs, Peterborough, UK). Spindle-shaped interstitial αSMA+ cells within the dermis were counted in six randomly chosen high-power fields by two observers in a blinded manner [35]. To image dermal collagen architecture by Second Harmonic Generation (SHG) microscopy [36], paraffin-embedded 10 μm thick skin sections were visualized using a multi-photon laser scanning upright microscope (Nikon A1 MP) tuned to 850 nm for two-photon excitation. SHG signal was collected in the transmission mode. 20x Nikon Plan Fluorite objective were used to focus the beam and the SHG light was collected with a 40x Nikon water immersion objective. The transmitted SHG signal was reflected by a dichroic mirror and separated from the fundamental beam, before detection by an intensified CCD camera. Digital images were captured at 512×512 pixels and averaged over two frames to improve the signal-to-noise ratio. Three randomly selected fields per mouse were analyzed using ImageJ (NIH) to quantify the SHG signal intensity representing the concentration and organization of collagen bundles. A lookup table pseudocolor scale correlation (Rainbow RGB scale, ImageJ) was applied to the images to highlight collagen deposition.

### Statistical analysis

Data are presented as means ± SD. Significance of differences between experimental and control groups was determined by Student's *t*-test. *p* < 0.05 was considered statistically significant. In the animal studies, differences between the groups were tested for their statistical significance by non-parametric Mann–Whitney *U* test.

## SUPPLEMENTARY MATERIALS FIGURES



## References

[1] Varga J, Abraham D (2007). Systemic sclerosis: a prototypic multisystem fibrotic disorder. J Clin Invest.

[2] Bhattacharyya S, Wei J, Varga J (2011). Understanding fibrosis in systemic sclerosis: shifting paradigms, emerging opportunities. Nat Rev Rheumatol.

[3] Wei J, Ghosh AK, Sargent JL, Komura K, Wu M, Huang QQ, Jain M, Whitfield ML, Feghali-Bostwick C, Varga J (2010). PPARgamma downregulation by TGFss in fibroblast and impaired expression and function in systemic sclerosis: a novel mechanism for progressive fibrogenesis. PLoS One.

[4] Fang F, Liu L, Yang Y, Tamaki Z, Wei J, Marangoni RG, Bhattacharyya S, Summer RS, Ye B, Varga J (2012). The adipokine adiponectin has potent anti-fibrotic effects mediated via adenosine monophosphate-activated protein kinase: novel target for fibrosis therapy. Arthritis Res Ther.

[5] Marangoni RG, Korman BD, Wei J, Wood TA, Graham LV, Whitfield ML, Scherer PE, Tourtellotte WG, Varga J (2015). Myofibroblasts in murine cutaneous fibrosis originate from adiponectin-positive intradermal progenitors. Arthritis Rheumatol.

[6] Ho YY, Lagares D, Tager AM, Kapoor M (2014). Fibrosis—a lethal component of systemic sclerosis. Nat Rev Rheumatol.

[7] Shupp A, Casimiro MC, Pestell RG (2017). Biological functions of CDK5 and potential CDK5 targeted clinical treatments. Oncotarget.

[8] Kawauchi T (2014). Cdk5 regulates multiple cellular events in neural development, function and disease. Dev Growth Differ.

[9] Pozo K, Bibb JA (2016). The Emerging Role of Cdk5 in Cancer. Trends Cancer.

[10] Liang Q, Li L, Zhang J, Lei Y, Wang L, Liu DX, Feng J, Hou P, Yao R, Zhang Y, Huang B, Lu J (2013). CDK5 is essential for TGF-beta1-induced epithelial-mesenchymal transition and breast cancer progression. Sci Rep.

[11] Bai X, Hou X, Tian J, Geng J, Li X (2016). CDK5 promotes renal tubulointerstitial fibrosis in diabetic nephropathy via ERK1/2/PPARgamma pathway. Oncotarget.

[12] Sargent JL, Milano A, Bhattacharyya S, Varga J, Connolly MK, Chang HY, Whitfield ML (2009). A TGFbeta-Responsive Gene Signature Is Associated with a Subset of Diffuse Scleroderma with Increased Disease Severity. J Invest Dermatol.

[13] Varga J, Marangoni RG (2017). Systemic sclerosis in 2016: Dermal white adipose tissue implicated in SSc pathogenesis. Nat Rev Rheumatol.

[14] Mori Y, Chen SJ, Varga J (2003). Expression and regulation of intracellular SMAD signaling in scleroderma skin fibroblasts. Arthritis Rheum.

[15] Mastrogiannaki M, Lichtenberger BM, Reimer A, Collins CA, Driskell RR, Watt FM (2016). beta-Catenin Stabilization in Skin Fibroblasts Causes Fibrotic Lesions by Preventing Adipocyte Differentiation of the Reticular Dermis. J Invest Dermatol.

[16] Chia JJ, Zhu T, Chyou S, Dasoveanu DC, Carballo C, Tian S, Magro CM, Rodeo S, Spiera RF, Ruddle NH, McGraw TE, Browning JL, Lafyatis R (2016). Dendritic cells maintain dermal adipose-derived stromal cells in skin fibrosis. J Clin Invest.

[17] Tsai LH, Delalle I, Caviness VS, Chae T, Harlow E (1994). p35 is a neural-specific regulatory subunit of cyclin-dependent kinase 5. Nature.

[18] Kim BS, Serebreni L, Fallica J, Hamdan O, Wang L, Johnston L, Kolb T, Damarla M, Damico R, Hassoun PM (2015). Cyclin-dependent kinase five mediates activation of lung xanthine oxidoreductase in response to hypoxia. PLoS One.

[19] Liebl J, Furst R, Vollmar AM, Zahler S (2011). Twice switched at birth: cell cycle-independent roles of the “neuron-specific” cyclin-dependent kinase 5 (Cdk5) in non-neuronal cells. Cell Signal.

[20] Tontonoz P, Hu E, Spiegelman BM (1994). Stimulation of adipogenesis in fibroblasts by PPAR gamma 2, a lipid-activated transcription factor. Cell.

[21] Shao X, Wang M, Wei X, Deng S, Fu N, Peng Q, Jiang Y, Ye L, Xie J, Lin Y (2016). Peroxisome Proliferator-Activated Receptor-gamma: Master Regulator of Adipogenesis and Obesity. Curr Stem Cell Res Ther.

[22] Banks AS, McAllister FE, Camporez JP, Zushin PJ, Jurczak MJ, Laznik-Bogoslavski D, Shulman GI, Gygi SP, Spiegelman BM (2015). An ERK/Cdk5 axis controls the diabetogenic actions of PPARgamma. Nature.

[23] Choi JH, Banks AS, Kamenecka TM, Busby SA, Chalmers MJ, Kumar N, Kuruvilla DS, Shin Y, He Y, Bruning JB, Marciano DP, Cameron MD, Laznik D (2011). Antidiabetic actions of a non-agonist PPARgamma ligand blocking Cdk5-mediated phosphorylation. Nature.

[24] Xie Z, Sanada K, Samuels BA, Shih H, Tsai LH (2003). Serine 732 phosphorylation of FAK by Cdk5 is important for microtubule organization, nuclear movement, and neuronal migration. Cell.

[25] Steinman RA, Robinson AR, Feghali-Bostwick CA (2012). Antifibrotic effects of roscovitine in normal and scleroderma fibroblasts. PLoS One.

[26] Mori Y, Ishida W, Bhattacharyya S, Li Y, Platanias LC, Varga J (2004). Selective inhibition of activin receptor-like kinase 5 signaling blocks profibrotic transforming growth factor beta responses in skin fibroblasts. Arthritis Rheum.

[27] Wei J, Melichian D, Komura K, Hinchcliff M, Lam AP, Lafyatis R, Gottardi CJ, MacDougald OA, Varga J (2011). Canonical Wnt signaling induces skin fibrosis and subcutaneous lipoatrophy: a novel mouse model for scleroderma?. Arthritis Rheum.

[28] Ghosh AK, Wei J, Wu M, Varga J (2008). Constitutive Smad signaling and Smad-dependent collagen gene expression in mouse embryonic fibroblasts lacking peroxisome proliferator-activated receptor-gamma. Biochem Biophys Res Commun.

[29] Zawel L, Dai JL, Buckhaults P, Zhou S, Kinzler KW, Vogelstein B, Kern SE (1998). Human Smad3 and Smad4 are sequence-specific transcription activators. Molecular cell.

[30] Ghosh AK, Bhattacharyya S, Lakos G, Chen SJ, Mori Y, Varga J (2004). Disruption of transforming growth factor beta signaling and profibrotic responses in normal skin fibroblasts by peroxisome proliferator-activated receptor gamma. Arthritis Rheum.

[31] Akhmetshina A, Palumbo K, Dees C, Bergmann C, Venalis P, Zerr P, Horn A, Kireva T, Beyer C, Zwerina J, Schneider H, Sadowski A, Riener MO (2012). Activation of canonical Wnt signalling is required for TGF-beta-mediated fibrosis. Nature communications.

[32] Wu M, Melichian DS, Chang E, Warner-Blankenship M, Ghosh AK, Varga J (2009). Rosiglitazone abrogates bleomycin-induced scleroderma and blocks profibrotic responses through peroxisome proliferator-activated receptor-gamma. Am J Pathol.

[33] Lakos G, Takagawa S, Chen SJ, Ferreira AM, Han G, Masuda K, Wang XJ, DiPietro LA, Varga J (2004). Targeted disruption of TGF-beta/Smad3 signaling modulates skin fibrosis in a mouse model of scleroderma. Am J Pathol.

[34] Woessner JF (1961). The determination of hydroxyproline in tissue and protein samples containing small proportions of this imino acid. Archives of biochemistry and biophysics.

[35] Distler JH, Jungel A, Huber LC, Schulze-Horsel U, Zwerina J, Gay RE, Michel BA, Hauser T, Schett G, Gay S, Distler O (2007). Imatinib mesylate reduces production of extracellular matrix and prevents development of experimental dermal fibrosis. Arthritis Rheum.

[36] Chen X, Nadiarynkh O, Plotnikov S, Campagnola PJ (2012). Second harmonic generation microscopy for quantitative analysis of collagen fibrillar structure. Nat Protoc.

