# Supplementary material for: The non-neuronal cyclin-dependent kinase 5 is a fibrotic mediator potentially implicated in systemic sclerosis and a novel therapeutic target
Source: Oncotarget. 2017 Dec 20;9(12):10294–306. doi: 10.18632/oncotarget.23516 (PMC5828185; doi:10.18632/oncotarget.23516)
Supplement: Supplementary file 1 [file oncotarget-09-10294-s001.pdf]

The non-neuronal cyclin-dependent kinase 5 is a fibrotic mediator potentially implicated in systemic sclerosis and a novel therapeutic target

SUPPLEMENTARY MATERIALS

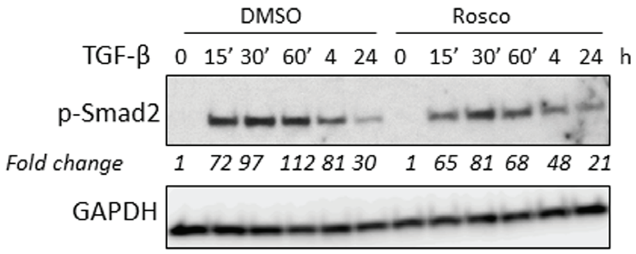

Supplementary Figure 1: Roscovitine does not block TGF-β-induced Smad2 phosphorylation. Fibroblasts were incubated in media with TGF-ß with or without roscovitine (10 µM) for indicated periods, and whole cell lysates were examined by Western analysis. Representative images. Band intensities normalized to input tubulin are shown.

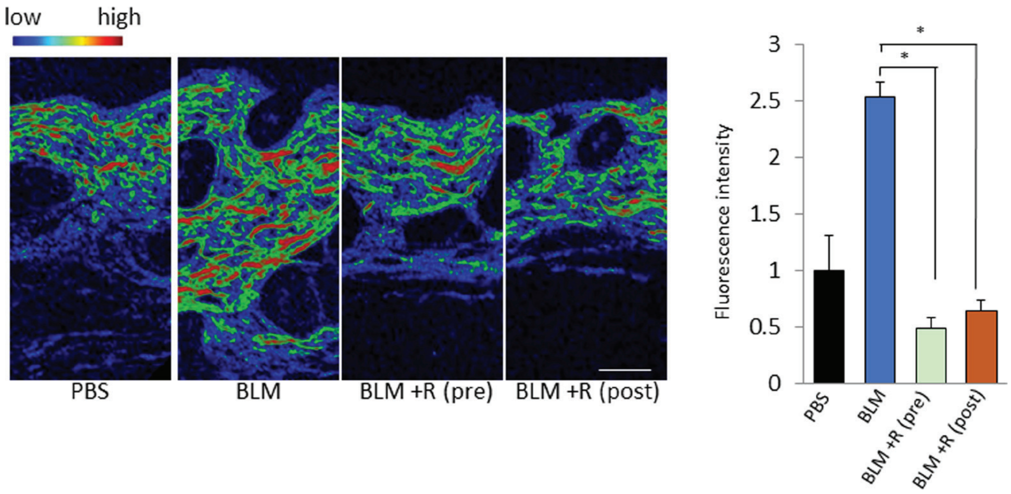

Supplementary Figure 2: Roscovitine prevents and reverses dermal collagen accumulation. Mice given s.c. bleomycin daily for 14 days, together with roscovitine (50 mg/kg/d i.p.) started concurrently with, or at day 15, of bleomycin, were sacrificed at day 28. Lesional skin was harvested and imaged by second harmonic generation (SHG) microscopy. Left, representative pseudocolor images. Right, relative intensity. SHG signal intensity, measured in three randomly selected fields per mouse using ImageJ (NIH). Scale bar, 50 µm. R, roscovitine.

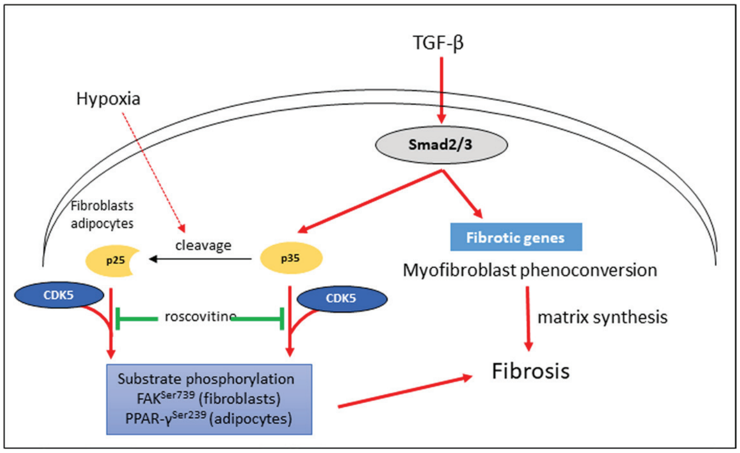

Supplementary Figure 3: Working model depicting the profibrotic activity of CDK5/p35. TGF-β elicits Smad-dependent p35 stimulation which results in augmented CDK5 activity. Hypoxia can also activate CDK5 signaling by inducing p35 expression and generation of the p25 cleavage product. Activated CDK5 in turn hyperphosphorylates FAK (Ser^{732}) which results in activation; and PPAR-γ (Ser^{239}) which results in reduced activity, with both contributing to profibrotic responses, including myofibroblast phenoconversion, epithelial-mesenchymal transition (EMT) and adipogenic-mesenchymal transition (AMT). By inhibiting CDK5 activity, roscovitine attenuates fibrotic TGF-ß responses.
